# Impact of an Online Sleep and Circadian Education Program on University Students’ Sleep Knowledge, Attitudes, and Behaviours

**DOI:** 10.3390/ijerph181910180

**Published:** 2021-09-28

**Authors:** Caitlin R. Semsarian, Gabrielle Rigney, Peter A. Cistulli, Yu Sun Bin

**Affiliations:** 1 Sleep Research Group, Charles Perkins Centre, University of Sydney, Camperdown, NSW 2006, Australia; caitlin.semsarian@sydney.edu.au (C.R.S.); peter.cistulli@sydney.edu.au (P.A.C.); 2 Appleton Institute of Behavioural Science, Central Queensland University, Wayville, SA 5034, Australia; g.rigney@cqu.edu.au; 3 Northern Clinical School, Sydney Medical School, University of Sydney, Camperdown, NSW 2006, Australia

**Keywords:** health education, health knowledge, attitudes, practice, sleep hygiene, chronobiology discipline, program evaluation, young adults, circadian rhythm

## Abstract

University students consistently report poor sleep. We conducted a before-and-after study to evaluate the impact of an online 10-week course on undergraduate students’ sleep knowledge, attitudes, and behaviours at 6-month follow-up. Data were collected via baseline course surveys (August–September 2020) and follow-up surveys distributed via email (February–March 2021). *n* = 212 students completed baseline surveys and *n* = 75 (35%) completed follow-up. Students retained to follow-up possessed higher baseline sleep knowledge and received higher course grades. At the 6-month follow-up, sleep knowledge had increased (mean score out of 5: 3.0 vs. 4.2, *p* < 0.001). At baseline, 85% of students aimed to increase their sleep knowledge and 83% aimed to improve their sleep. At follow-up, 91% reported being more knowledgeable and 37% reported improved sleep. A novel Stages of Change item revealed that 53% of students’ attitudes towards their sleep behaviours had changed from baseline. There was a reduction in sleep latency at follow-up (mean 33.3 vs. 25.6 min, *p* = 0.015), but no change in the total Pittsburgh Sleep Quality Index score. In summary, completion of an online course led to increased sleep and circadian knowledge and changed sleep attitudes, with no meaningful change in sleep behaviours. Future interventions should consider components of behavioural change that go beyond the knowledge–attitudes–behaviour continuum.

## 1. Introduction

Sleep is vital for maintaining optimum health and quality of life. For young adults (18–25 years), sleep is particularly crucial for facilitating academic success [1,2,3]. Key components of learning capacity such as neurocognitive processing, procedural learning, and declarative learning are all impaired with insufficient sleep [4]. Furthermore, cognitive barriers to academic success are often compounded by sleep-related psychiatric disorders such as depression, anxiety, and substance use disorders [5]. These findings are of serious concern, because approximately 60% of college students report poor sleep quality, 27% are at risk of at least one sleep disorder, and rates of insomnia range from 9.4% to 38.2% [6,7,8]. Lifestyle factors that contribute to the exceptionally poor sleep health of this group include increased screen time, increased substance use, and circadian disruption due to the discrepancy between academic commitments and social schedules [9]. Furthermore, students with evening chronotypes (i.e., their circadian rhythms produce peak alertness in the evening [10]) have been shown to receive poorer academic results [11] and may be at a greater risk of depressive symptoms than their morning-type and intermediate chronotype counterparts [12].

Considering what is known about young adult sleep behaviours, few university campuses across the world have delivered sleep education programs to their students. However, despite the wide-reaching potential of such programs, three recent systematic reviews identified only a total of 17 sleep education interventions for university students [13,14,15]. Of these 17 interventions, most comprised didactic sessions in which students were informed of a range of behavioural and environmental modifications that promoted sleep (i.e., provided sleep hygiene advice). Follow-up periods were brief and ranged from immediately post-intervention to three months. Although some interventions led to increased sleep knowledge among students [16,17,18], the impact on sleep behaviours was highly variable, with few studies demonstrating changes in self-reported sleep latency [17] and sleep hygiene [18,19]. Ultimately, only one study demonstrated an increase in total Pittsburgh Sleep Quality Index (PSQI) scores [19]. Eight interventions utilised online methods of content delivery. The efficacy of online interventions has repeatedly been demonstrated in online cognitive–behavioural therapy programs for adults with insomnia, and represents a cost-effective and accessible method of program delivery [20,21].

Furthermore, few interventions incorporated behavioural change theory in their intervention design, or considered students’ preparedness to change their sleep behaviours [15]. Psychologists recommend embedding behavioural change models within sleep education initiatives in order to understand the gap between the dissemination of health information and improved health behaviours [22]. Although multiple models of behavioural change exist, common to many is a continuum between the acquisition of knowledge, changes in attitudes, and behavioural modification [23]. Designs and evaluations of previous sleep education interventions for young adults have often focused on capturing changes in sleep behaviours and/or sleep quality alone, without simultaneous consideration of students’ sleep knowledge or attitudes [13,14]. By intentionally facilitating and capturing shifts in sleep knowledge and attitudes, educators can better foster movement towards healthier sleep behaviours for all students no matter their existing sleep practices. Therefore, this before-and-after study sought to determine whether completion of an interactive and online educational course on sleep and circadian rhythms improves (1) sleep knowledge, (2) attitudes towards changing sleep behaviours, and (3) sleep behaviours in university students at the 6-month follow-up.

## 2. Materials and Methods

‘Health Challenges: Sleep’ is an online course on sleep and circadian health available to students at the University of Sydney. It was developed as an elective subject for the general education of undergraduate students, and therefore assumes no prior knowledge. The aims of developing the course were to remedy the lack of education on sleep and circadian rhythms in the Australian school system, and to provide this information at a tertiary level in an integrated fashion for practical use and understanding, rather than as a higher-level (senior) subject in specific disciplinary areas such as neuroscience, psychology, and physiology.

The course was delivered via the university’s online learning management system (Canvas) and is intended to require 40–50 h of student effort across 8 modules and a final assessment. The 8 modules of the course are: (1) Introduction to Sleep, (2) Introduction to Circadian Rhythms, (3) Sleep Deprivation, (4) Jetlag, (5) Shift Work, (6) Measurement of Sleep and Circadian Rhythms, (7) Sleep Hygiene and Insomnia, and (8) Other Common Sleep and Circadian Disorders [24]. Potential participants in the current study were 379 students who enrolled and completed the unit in 2 Semester 2020 (during the 10 weeks from 24th August to 8th November). Each module was completed at the students’ leisure to weekly deadlines, with two weeks provided for the final assessment.

Components of the unit designed to facilitate behavioural change included:

- To increase knowledge: practical advice including the impact of light/darkness on delaying or advancing circadian rhythms, napping, and caffeine, sleep environment recommendations, and other sleep hygiene tips to enhance sleep quality were embedded within course materials;
- To encourage self-reflection: self-monitoring of sleep using a sleep diary, use of an app-based lux meter to measure light levels outdoors under sunlight compared to indoor artificial lighting compared to darkness, student completion of a novel Stages of Change item to identify existing attitudes towards changing sleep behaviours, participation in surveys about sleep behaviours and sleep quality, with results of surveys fed back to students to allow comparison with cohort behavioural norms;
- To increase motivation for behavioural change: students were asked to share motivations for enrolling in the course via the online discussion board, and students selected outcome goals upon commencing the course;
- To encourage application of course content: structured discussion activities in which students were encouraged to share and analyse their own sleep habits and environments; and the final assessment comprised an advocacy-based task where students were required to write a letter to a leader in the community (e.g., high school principal) outlining how the sleep and circadian health of that community (e.g., high school students) can be improved.

### 2.1. Baseline Data Collection

The following data were collected through surveys distributed during the course (beginning of 2 Semester 2020).

- Demographic information: Student ID, gender, age, hours of previous sleep education, shift work completed in the previous month, and chronotype was collected. Chronotype was determined via completion of the Morningness–Eveningness Questionnaire [10]. The standard scores were used to classify students into evening-types (16–41), intermediate (42–58), and morning-types (59–86);
- Sleep knowledge: Prior to starting the course, students were asked five multiple-choice questions regarding key sleep concepts. The questions tested key concepts taught in the course. Of the five questions, three questions tested practical concepts relevant to the young adult age group, and two questions tested theoretical concepts in the two-process model of sleep regulation;
- Sleep attitudes: This question was modelled on the Stages of Change model of behavioural change [25], with each option representing one of the stages of change. Students were asked “Which statement best describes your current attitudes towards your sleep?”, with possible answers being “I am not interested in changing my current sleeping habits” (precontemplation), “I would consider making lifestyle changes to improve my sleep” (contemplation), “I am seeking information and gathering resources in order to improve my sleep” (preparation), “I am currently practicing lifestyle changes in order to improve my sleep” (action), “I have been using lifestyle changes to foster good sleep for at least 6 months now” (maintenance) and “I cannot change my current sleeping habits” (relapse). Although no standardised stages of change questions have been developed for use in sleep health promotion, various single-question items have been trialled for physical activity promotion [26];
- Student goals: Students were asked “What do you hope to achieve upon completing this course? (Select all that apply)”, with possible answers being “Complete 2 credit points of study”, “Increase my knowledge of sleep”, “Improve or change my sleep habits” and “Other (please specify)”;
- Sleep behaviours: Sleep behaviours were assessed using two scales: the Sleep Hygiene Index (SHI) [27] and the Pre-Bed Behaviour Questionnaire (PBBQ) [28]. Items on both scales were modified to remove duplication and to reflect contemporary uses of technology (Supplementary Figure S1). The SHI comprises 13 items, of which 8 were used in the current study to assess the frequency of unhealthy sleep behaviours. The original was developed in a university student sample and has been shown to have good internal consistency (Cronbach’s alpha = 0.66) and good test–retest reliability (r = 0.71) [27]. The original PBBQ comprised 25 items and was designed to capture common behaviours that occur in the hour prior to sleeping and was developed in high school students [28]. In the current study, the PBBQ items were simplified to 15 items by removing items that were already captured by the SHI and combining others (e.g., watching TV and watching DVDs). For both scales, the more frequent the behaviours, the poorer the sleep hygiene, and the higher the global score;
- Caffeine intake: Students were asked “On an average day, how many of the following caffeinated beverages do you usually drink?” Caffeinated beverages listed were a 250 mL energy drink, 375 mL can of cola drink, 250 mL cup of tea, 250 mL cup of coffee, short black/espresso coffee, and other. Possible answers were “0/1/2/3/4/5+.” The quantity (mg) of caffeine consumed daily was calculated from their responses [29];
- Light exposure: Light exposure was queried using three questions. These were, “On an average day, how many hours do you usually spend outdoors?”, “How long are you awake in the morning until you see sunlight? For example, opening your window or going outside.” and “Do you use any software or apps to adjust the colour display on your smartphone, laptop, or other computing devices? These might include Night Shift (Mac), Night Light (Windows), or f.lux (application)” with responses being “No/On some of my devices/On all of my devices”;
- Sleep quality: Students completed the 19-item Pittsburgh Sleep Quality Index (PSQI). The PSQI is a widely used measure of sleep quality which provides a total score (with a possible total of 21) derived from the evaluation of seven components of sleep (quality, latency, duration, efficiency, disturbance, use of sleep medications, and daytime dysfunction) over the previous month [30]. The PSQI has high internal consistency (Cronbach’s alpha 0.76), moderate test–retest reliability (r = 0.64) and is modestly correlated with objective measures of sleep (r = 0.2 to 0.5) [31]. The total scores were used to categorise students into good sleepers (≤5) and poor sleepers (>5).

### 2.2. Follow-Up Data Collection

*n* = 212 students enrolled in the course completed the baseline surveys (55.9% of total enrolled students). All were invited to participate in the follow-up survey by email on 23 February 2021, approximately 6 months after the baseline data collection at the beginning of the course. Up to three reminder emails were sent to non-responders on 1, 4, and 15 March 2021 (beginning of 1 Semester 2021).

The follow-up survey included a repeat of the baseline data collection, and the following measure:

- Student reflection: Students were asked “Upon completing the course, did you achieve the goals you set at the beginning of the course? (Select all that apply)” with possible answers being “I completed 2 credit points of study,” “I am more knowledgeable about sleep,” “I have changed my sleep habits as a result,” “My sleep has changed as a result,” and “Other.”

Student performance in the course was obtained from the online learning management system.

### 2.3. Ethics Approval

Ethical approval for this study was provided by the University of Sydney Human Research Ethics Committee (Project Number: 2020/089). Completion of all surveys was voluntary, and participants gave informed consent to the use of their data for research purposes. Students were offered an AUD 5 voucher or direct reimbursement for return of the follow-up survey.

### 2.4. Quantitative Analysis

Respondents and non-respondents were compared to evaluate the representativeness of students who completed the follow-up. Chi-squared tests were used to detect differences in proportions for categorical variables (gender, amount of prior sleep education, baseline PSQI sleeper type, chronotype, shift work completed in the past month), Fisher’s exact test was used where there were cell sizes smaller than 5 (age groups), and independent-samples *t*-tests were used to test for differences in the means of continuous variables (mark in course, baseline sleep knowledge score, baseline PSQI total score).

For the students who completed the follow-up survey, descriptive statistics were used to illustrate student goal-setting, goal achievement, and sleep attitudes. Paired *t*-tests were used to compare differences in continuous variables (sleep knowledge score, sleep behaviour questionnaire scores, caffeine intake, hours awake before seeing sunlight, hours spent outdoors, PSQI total score). Wilcoxon signed-rank tests were used to compare differences in categorical variables (Stages of Change question responses, use of blue light filters, PSQI subcomponent scores) before and after the course.

All statistical analyses were carried out using IBM SPSS Statistics 27 for Macintosh (Armonk, NY, USA: IMB Corp).

## 3. Results

### 3.1. Representativeness of Follow-Up

*n* = 212 students enrolled on the course fully completed the baseline survey. The characteristics of students who completed the baseline survey only (*n* = 137), compared to those who additionally completed the follow-up survey (*n* = 75, 35%) are depicted in Table 1. Median age, gender distribution, amount of prior sleep education, chronotype distribution, and proportion of students who completed shift work in the past month were similar between non-responders and follow-up students (Table 1). However, respondents received significantly higher marks in the course (83.1 vs. 87.1, *p* = 0.004) and had higher baseline sleep knowledge (mean score out of 5: 2.6 vs. 3.0, *p* = 0.005) despite having a similar amount of prior sleep education (Table 1). Although baseline PSQI scores were comparable between the two groups (6.2 vs. 5.9), the groups of students who completed the follow-up survey had a higher proportion of good sleepers at baseline (42 vs. 57%, *p* = 0.037).

### 3.2. Sleep Knowledge

For the respondents, there was a significant increase in sleep knowledge before and 6 months after completion of the course. The mean score out of 5 was 3.0 (SE 0.1) at baseline vs. 4.2 (SE 0.1) at the 6-month follow-up (t_74_ = −9.178, *p* < 0.001). Sleep concepts addressed and responses are depicted in Table 2.

### 3.3. Sleep Attitudes

At baseline, 85% of students aimed to increase their knowledge about sleep, and 83% aimed to change or improve their sleep habits. At the 6-month follow-up, 91% reported that they were more knowledgeable about sleep, 35% reported that they had changed their sleep habits, and 37% reported that their sleep had improved upon completion of the course.

**Figure 1:**
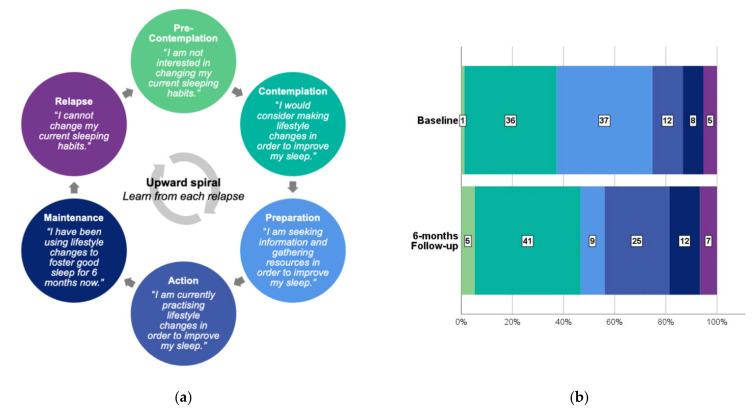
(**a**) Stages of Change Model of behavioural change [25]. (**b**) Students’ (*n* = 75) attitudes towards sleep behaviours before and 6 months after completion of a sleep and circadian education course.

Regarding the Stages of Change model of behavioural change, there was no significant difference in the distribution of responses before and after course completion (z = −0.805, *p* = 0.421). The majority (53%) of students selected different phases at baseline and follow-up. At baseline, 37% of students were in the preparation phase of behavioural change. At the 6-month follow-up, only 9% of students remained in the preparation phase. There were increases in the proportion of students in all other groups at the 6-month follow-up, i.e., contemplation (36% vs. 41%), action (12% vs. 25%), maintenance (8% vs. 12%), relapse (5% vs. 7%), and pre-contemplation (1% vs. 5%). These attitudes towards changing sleep behaviours are depicted in Figure 1.

### 3.4. Sleep Behaviours

Table 3 summarises the sleep and circadian behaviours of followed-up students. Significant changes included a decrease in the modified Sleep Hygiene Index total score indicating an improvement in sleep hygiene (mean score/32: 12.4 vs. 11.4, *p* = 0.040), and decreased time awake prior to seeing sunlight (mean 0.6 vs. 0.4 h, *p* = 0.042). At follow-up, daily caffeine intake had significantly increased (mean 69.7 vs. 83.8 mg, *p* = 0.026) and there was a significant trend towards the decreased use of blue light filters on electronic devices (*p* = 0.014). There were no significant changes in the modified Pre-Bed Behaviour Questionnaire score, or hours spent outdoors. The proportions of students endorsing each item on the modified Sleep Hygiene Index and Pre-Bed Behaviour Questionnaire surveys are depicted in Supplementary Figure S1a,b. There was non-significant movement towards less variable bedtimes, less excessive time in bed, and less time spent thinking, planning, or worrying before bed.

PSQI scores collected before and 6 months after course completion are depicted in Table 4. There were no statistical differences in the total PSQI score (mean difference −0.07; 95% CI −0.75, 0.62) or in the proportion of students categorised as good sleepers. No sub-components of the PSQI were significantly altered except for sleep latency and waketime. There was shorter sleep latency, with 49% reporting falling asleep in less than 15 min after the course compared to 44% at baseline. Waketimes also occurred earlier without a corresponding change in bedtimes.

## 4. Discussion

We found evidence of increased sleep knowledge, altered attitudes towards changing sleep behaviour, and some changes in sleep hygiene, with no overall change in total PSQI scores 6 months following an online sleep and circadian education course for undergraduate students. These results are highly consistent with previous sleep education interventions. Previous interventions have demonstrated increased sleep knowledge at up to 10 weeks post-intervention [16,17,18], with this study being the first to demonstrate sustained increases in knowledge at the 6-month follow-up. This result is unsurprising, because the intervention was a university course which is optimised for information dissemination.

Regarding attitudes towards sleep, this is the first study to explicitly assess attitudes towards sleep behaviour modification. Although the overall results were not statistically significant, attitudes captured via a novel Stages of Change item revealed that 37% of students were preparing to change their sleep at baseline compared to only 9% of students at follow-up. The proportion of students who were actively changing their sleep habits sleep habits had doubled. Furthermore, 35% of students reported that they had changed their sleep habits, suggesting that there may have been a group of students who progressed from preparation to action phases according to the Stages of Change model. The only other study which has examined attitudes demonstrated a decrease in maladaptive beliefs about sleep at a 4-week follow-up [17].

Our intervention resulted in minor changes in sleep behaviour, despite 35% of students reporting that they had changed their sleep habits. Although there was a slight improvement in Sleep Hygiene Index scores and earlier exposure to sunlight upon waking, caffeine intake increased, and the use of blue light filters decreased. Both the baseline and follow-up surveys were completed at the beginning of respective semesters, thereby reducing semester/vacation variation in students’ activities. However, increased caffeine intake may reflect a broader trend of increasing caffeine intake throughout university [32]. Regardless, caffeine intake remained below the maximum recommended daily limit (400 mg). The timing of the caffeine was not recorded, but should be in future studies, because the timing of caffeine intake has a greater influence on sleep [29]. Little is known about the uptake and patterns of blue light filters among any population, but the majority of students in the current study appeared to already be using blue light filters on electronic screens. There was a significant trend towards earlier waketimes with no corresponding change in bedtimes, which may also suggest that university students’ sleep patterns are highly influenced by environmental circumstances. These results are consistent with the literature, with interventions showing limited effects on sleep hygiene [16,33], self-reported improvements in sleep habits [18], and improvements in Sleep Hygiene Index scores [19].

Given that there were minimal changes in sleep behaviours, it is unsurprising that there was no change in total PSQI score at the 6-month follow-up. This is consistent with the literature, where only one study has reported an improvement in total PSQI scores [19]. Unfortunately, this finding has not been replicated by similar interventions, but suggests that interventions of shorter duration, lower intensity, and lower cost may still be effective. Interestingly, despite no change in PSQI scores, 37% of students reported improved sleep at follow-up. Potential explanations include a placebo effect due to the time spent completing this course, a perceived improvement due to decreased sleep latencies, and a perceived improvement when students compared their own behaviours to the sleep of the whole cohort.

Ultimately, we must consider why this educational intervention did not lead to any meaningful changes in sleep behaviour or quality, despite demonstrating students’ knowledge retention and willingness to improve their sleep. It may be concluded that as with other sleep education interventions, this course remained predominantly an educational program, rather than a health promotion initiative.

Future iterations of this course could be designed with a greater emphasis on behavioural change. Firstly, the use of existing course components could be expanded. For example, sleep diary use could be extended because ongoing self-monitoring is more likely to lead to behavioural change [34]. Additionally, baseline student goals could be adapted to a S.M.A.R.T. (specific, measurable, attainable, realistic/relevant, timed) goal-setting activity; these are commonly used in behavioural change interventions [35,36]. Secondly, the design of future interventions could be guided by newer theories of behavioural change that extend beyond the traditional knowledge–attitudes–behaviour model. This model fails to consider students’ individual contexts and abilities, which is relevant because the contextual barriers to good sleep hygiene among university students are well-described [9]. The knowledge–attitudes–behaviour model also fails to consider student motivation, which is a key component of behavioural change that could be promoted via activities such as motivational interviewing. An example of a newer model of behavioural change that addresses the limitations of the knowledge–attitudes–behaviour model and which could guide future course development is the COM-B model, where altered behaviour is the result of sufficient capability, opportunity, and motivation [37]. Finally, novel strategies to encourage and maintain behavioural change should be considered, such as sleep challenges where students are tasked with trialling a specified sleep hygiene strategy, or a behaviour known to improve circadian rhythmicity [38]. The main challenge in educational programs such as this one is integrating health promotion initiatives to align with both the goals of education and health promotion.

Although education provides the knowledge that is necessary for behaviour change, changes in behaviour do not occur on the basis of knowledge gains alone. Effective health promotion provides individuals with the tools and the skills to implement and sustain changes in behaviour, and it is these elements that are missing from the current intervention, and which should be added in future iterations. However, given the limited sleep education for children and adolescents in Australia and the lack of sleep health coverage in public health programs, the major advantage of the intervention is that it provides the necessary first step in promoting sleep and circadian health.

Strengths of this study include the simultaneous measurement of sleep knowledge, attitudes, and behaviours, extended follow-up period, increased intervention duration, and engaging design, and inclusion of health-promoting components in the intervention itself. Furthermore, because the course comprised online modules that could be completed at any time, we expect that all students, regardless of chronotype, would be able to complete the course at a time that suited them. This may overcome the circadian asynchrony caused by university schedules which is hypothesised to account for the poorer academic performance of students with evening chronotypes [11].

The main limitation of this study is the before-and-after study design and lack of a control group. This study was also limited by a low response rate (35%). Although the response rate was comparable to general online survey response rates, future interventions should consider novel methods to incentivise participation [39]. With increased participation, further analyses such as evaluations of whether chronotype and other participant characteristics drive the effectiveness of sleep education would be feasible and provide further insights into how the intervention could be tailored and enhanced. The impact of this intervention may have also been limited by the sample population. Students who elected to complete this course may already demonstrate higher levels of health-seeking behaviours. Furthermore, those students who were retained at follow-up possessed higher baseline sleep knowledge, and 57% were classified as good sleepers at baseline, thereby minimising the potential overall effect on sleep quality.

Future evaluations require the use of more comprehensive and validated measures. For example, sleep knowledge was assessed with five multiple-choice questions on various concepts chosen by the authors, but could be assessed with a more comprehensive survey that is known to be correlated with sleep quality, such as the Sleep Knowledge Questionnaire [40]. Insights into students’ attitudes towards sleep that predispose towards insomnia could be better captured through use of the Dysfunctional Beliefs About Sleep scale [41], and the Stage of Change item could be formally assessed for validity. For behaviours such as the use of electronic screens, distinguishing between the various types of screen use (e.g., study/employment vs. recreational) will also better characterise the patterns of blue light exposure in daily life, and may also identify potential opportunities for sleep hygiene interventions. Finally, all data were subjectively reported by students. In future, more extensive use of sleep diaries accompanied by the use of actigraphy, which can provide both objective measures of sleep and of light exposure, would be ideal for substantiating the self-reported changes.

## 5. Conclusions

In conclusion, an online sleep and circadian education course led to increased sleep knowledge and changed attitudes with no significant change in sleep behaviours or sleep quality among university students at the 6-month follow-up. Future interventions require careful design and evaluation and should consider components of behavioural change that go beyond the knowledge–attitudes–behaviour continuum.

## Figures and Tables

**Table 1:** Characteristics of students who completed the baseline survey only, and those who additionally completed the follow-up survey.

	Non-Responders *n* = 137 *n* (%)	Responders *n* = 75 *n* (%)	Test Statistic, *p*-Value
**Age** median(IQR)	20.0 (19.0–20.0)	19.0 (18.0–20.0)	Fisher’s exact, *p* = 0.271
17–19 years	68 (50)	46 (61)	
20–24 years	65 (47)	27 (36)	
25+ years	4 (3)	2 (3)	
Gender *			χ^2^_1_ = 0.087, *p* = 0.768
Male	37 (27)	19 (25)	
Female	99 (72)	56 (75)	
**Mark out of 100** mean(SD)	83.1 (10.3)	87.1 (8.4)	t_210_ = −2.891, *p* = 0.004
**Amount of prior sleep education**			χ^2^_2_ = 4.395, *p* = 0.111
0 h	92 (67)	59 (79)	
1–5 h	23 (17)	11 (15)	
≥6 h	22 (16)	5 (6)	
**Baseline sleep knowledge score** mean out of 5(SD)	2.6 (1.0)	3.0 (1.0)	t_210_ = −2.810, *p* = 0.005
**Baseline PSQI total score** (mean out of 21, SD)	6.2 (2.6)	5.9 (3.3)	t_210_ = 0.823, *p* = 0.412
**Baseline PSQI categories**			χ^2^_1_ = 4.370, *p* = 0.037
Good sleeper (PSQI ≤ 5)	58 (42)	43 (57)	
Poor sleeper (PSQI > 5)	79 (58)	32 (43)	
**MEQ**			χ^2^_2_ = 2.028, *p* = 0.363
Evening type (16–41)	44 (32)	21 (28)	
Intermediate (42–58)	71 (52)	36 (48)	
Morning type (59–86)	22 (16)	18 (24)	
**Shift work in the last month**	35 (26)	15 (20)	χ^2^_1_ = 0.828, *p* = 0.363

IQR: interquartile range. MEQ: Morningness-Eveningness Questionnaire. PSQI: Pittsburgh Sleep Quality Index. SD: standard deviation. * 1 missing response in the non-responder group. Fisher’s exact test was used for categorical variables where there were cell sizes <5. Otherwise, categorical variables were analysed with chi-squared tests, and continuous variables were analysed with independent *t*-tests.

**Table 2:** Key concepts assessed via multiple-choice questions before and 6 months after the completion of a sleep and circadian education course (*n* = 75).

Sleep Concept Assessed	% Correct	
	Baseline	6-Month Follow-Up
Two-process model of sleep regulation	88	96
Influence of morning light on phase advancement	64	93
Recommended sleep duration for young adults (18–25 years old)	63	75
Circadian delay in teenagers	56	77
Influence of napping on drive to sleep	27	79

**Table 3:** Sleep behaviours before and 6 months after completion of a sleep and circadian education course (*n* = 75).

Characteristic	Baseline	6-Month Follow-Up	Test Statistic, *p*-Value
**Modified Sleep Hygiene Index** mean out of 32(SD)	12.4 (4.3)	11.4 (4.9)	t_74_ = 2.090, *p* = 0.040
**Modified Pre-Bed Behaviour Questionnaire** mean out of 45(SD)	15.5 (4.6)	15.4 (5.4)	t_74_ = 0.135, *p* = 0.893
**Daily caffeine intake** (mg) mean (SD)	69.7 (71.9)	83.8 (78.0)	t_74_ = −2.276, *p* = 0.026
**Hours awake before seeing sunlight** mean (SD)	0.6 (0.7)	0.4 (0.7)	t_74_ = 2.073, *p* = 0.042
**Hours spent outdoors** mean (SD)	2.3 (1.7)	2.3 (1.9)	t_74_ = −0.291, *p* = 0.772
**Use of blue light filters on devices***n* (%)			z = −2.466, *p* = 0.014
No	12 (16)	26 (35)	
On some of my devices	29 (39)	18 (24)	
On all of my devices	34 (45)	31 (41)	

SD: standard deviation.

**Table 4:** Pittsburgh Sleep Quality Index (PSQI) scores and sub-components before and 6 months after completion of a sleep and circadian education course (*n* = 75).

Characteristic	Baseline *n* (%)	6-Month Follow-Up *n* (%)	Test Statistic, *p*-Value
**PSQI total score** mean out of 21(SD)	5.9 (3.3)	6.0 (3.2)	t_74_ = −0.194, *p* = 0.847
Good sleep (≤5)	43 (57)	41 (55)	z = −0.471, *p* = 0.637
Poor sleep (>5)	32 (43)	34 (45)	
**Sleep duration**			z = −1.237, *p* = 0.216
≤6 h	8 (11)	13 (17)	
7 h	19 (25)	15 (20)	
8 h	24 (32)	29 (39)	
≥9 h	24 (32)	18 (24)	
**Sleep quality**			z = −0.554, *p* = 0.580
Very good	13 (17)	9 (12)	
Fairly good	45 (61)	51 (68)	
Fairly bad	16 (21)	13 (17)	
Very bad	1 (1)	2 (3)	
**Sleep latency**			z = −2.118, *p* = 0.034
0–15 min	33 (44)	37 (49)	
16–30 min	24 (32)	27 (36)	
31–60 min	11 (15)	6 (8)	
>60 min	7 (9)	5 (7)	
**Sleep efficiency**			z = −0.191, *p* = 0.849
≥85%	51 (68)	50 (67)	
75%–84%	13 (17)	13 (17)	
65%–74%	6 (8)	7 (9)	
<65%	5 (7)	5 (7)	
**Bedpartners**			z = −0.975, *p* = 0.330
No bedpartner	55 (73)	55 (73)	
Partner/roommate in other room	2 (3)	8 (11)	
Partner in same room but not same bed	6 (8)	3 (4)	
Partner in same bed	12 (16)	9 (12)	
**Sleep medication use**			z = −1.382, *p* = 0.167
Not during the past month	70 (94)	71 (95)	
Less than once a week	1 (1)	3 (4)	
Once or twice a week	1 (1)	1 (1)	
Three or more times a week	3 (4)	0 (0)	
**Bedtime** median(IQR)	23:59 (22:40–02:00)	24:00 (23:00–02:00)	z = −0.828, *p* = 0.408
**Waketime** median(IQR)	9:00 (7:45–10:30)	9:00 (7:30–10:00)	z = −2.494, *p* = 0.013
**Midpoint of sleep** median(IQR)	04:15 (03:06–06:00)	04:19 (03:14–05:30)	z = −1.340, *p* = 0.180

IQR: interquartile range. PSQI: Pittsburgh Sleep Quality Index. SD: standard deviation. Continuous variables were analysed with paired *t*-tests. Categorical variables were analysed with Wilcoxon signed-rank tests.

## Data Availability

The data presented in this study are available on request from the corresponding author. The data are not publicly available due to ethical considerations.

## Supplementary Materials

The following are available online at https://www.mdpi.com/article/10.3390/ijerph181910180/s1, Figure S1: (a) Modified Sleep Hygiene Index, (b) Modified Pre-Bed Behaviour Questionnaire.
