# Supplementary material for: Impact of an Online Sleep and Circadian Education Program on University Students’ Sleep Knowledge, Attitudes, and Behaviours
Source: Int J Environ Res Public Health. 2021 Sep 28;18(19):10180. doi: 10.3390/ijerph181910180 (PMC8507829; doi:10.3390/ijerph181910180)
Supplement: Supplementary file 1 [file ijerph-18-10180-s001.zip › ijerph-1365363-supplementary.pdf]

International Journal of
Environmental Research and Public Health

MDPI

Supplementary

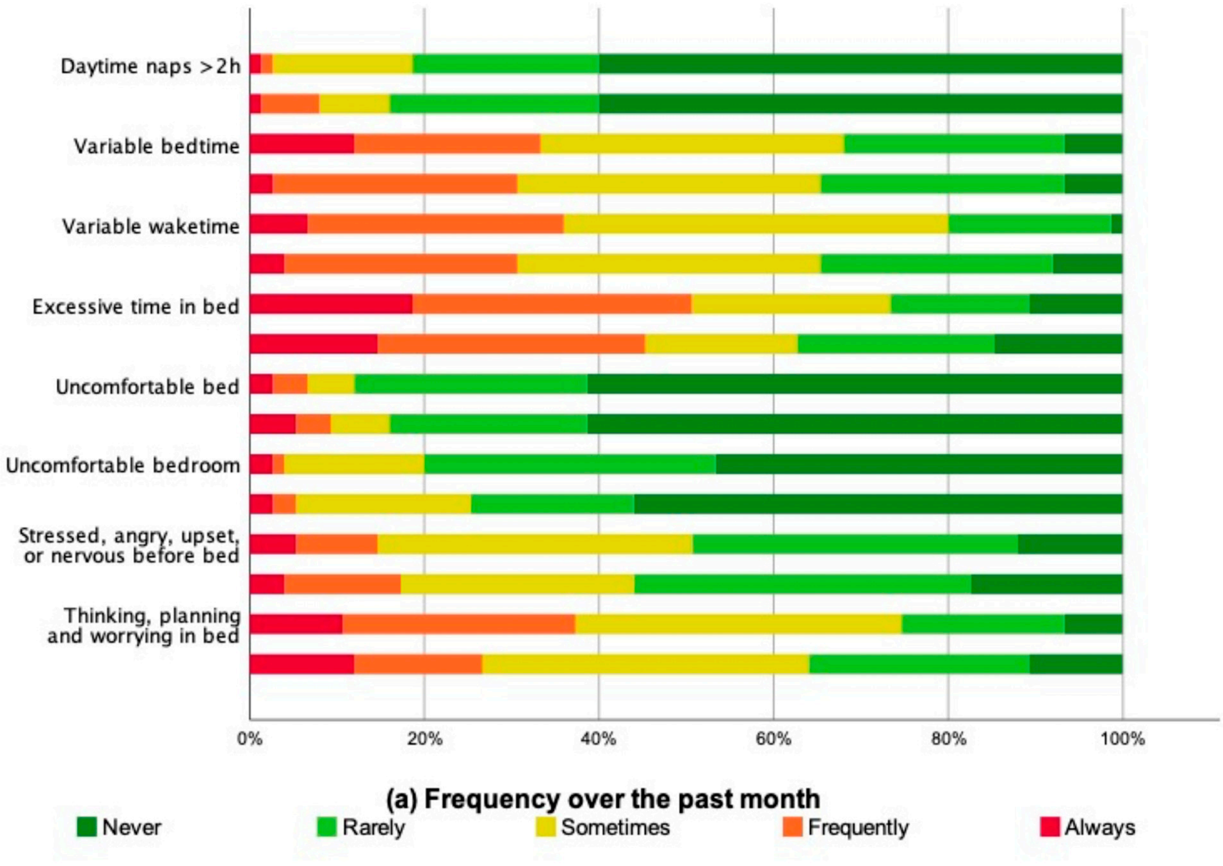

Figure S1. (a) Modified Sleep Hygiene Index. Proportion of students endorsing items on the modified Sleep Hygiene Index (n = 75) before and 6-months after completion of a sleep and circadian education course. For each pair of bars, the upper bar represents survey responses at baseline and the lower bar represents survey responses at 6-months follow-up.

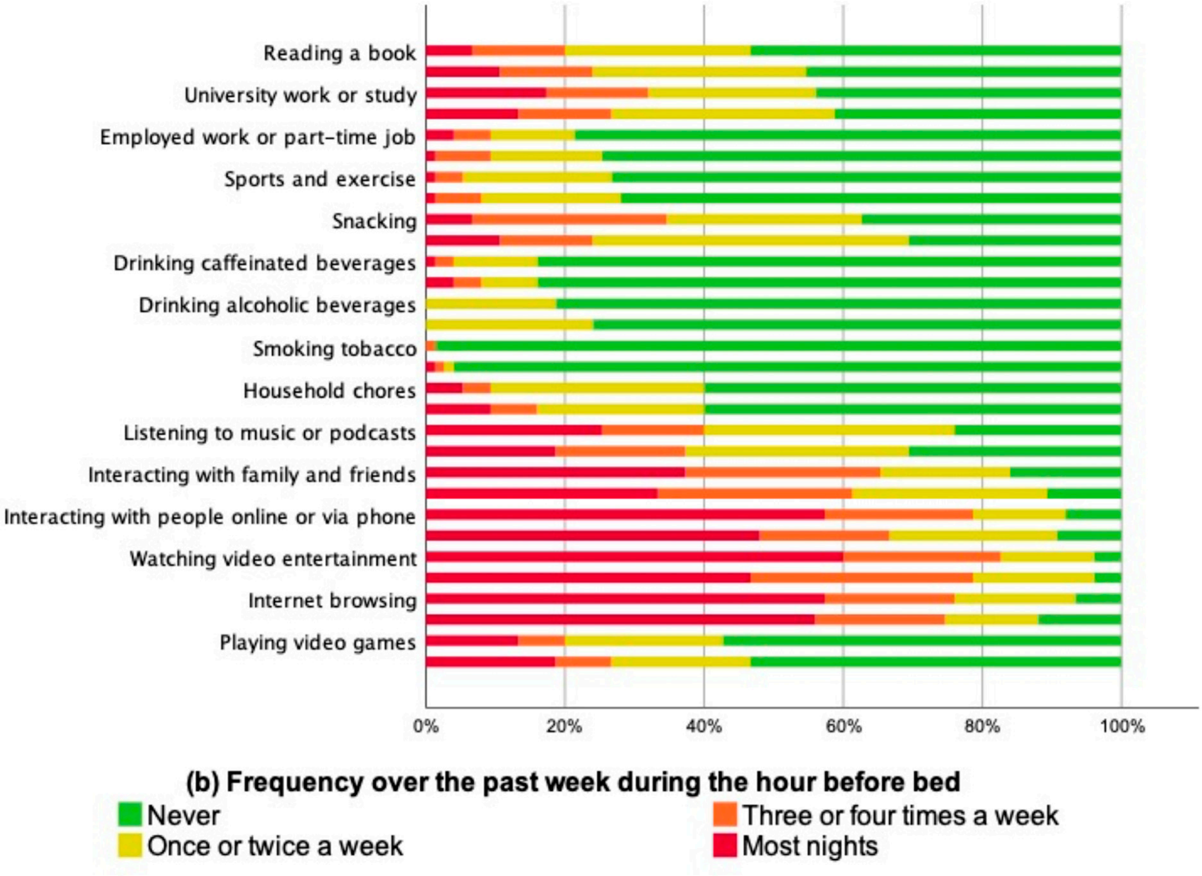

Figure S1. (b) Modified Pre-Bed Behaviour Questionnaire. Proportion of students endorsing items on the Pre-Bed Behaviour Questionnaire (n = 75) before and 6-months after completion of a sleep and circadian education course. For each pair of bars, the upper bar represents survey responses at baseline and the lower bar represents survey responses at 6-months follow-up.
